# Internal jugular vein vascular malformation presenting as mass at root of neck: a case report

**DOI:** 10.1186/1472-6815-9-5

**Published:** 2009-06-14

**Authors:** Prahlad Duggal, Pankaj Chaturvedi, Prathamesh S Pai, Deepa Nair, SL Juvekar, Bharat Rekhi

**Affiliations:** 1 Head and Neck Services, Department of Surgical Oncology, Tata Memorial Hospital, Parel, Mumbai, India; 2 Department of Radiodiagnosis, Tata Memorial Hospital, Parel, Mumbai, India; 3 Department of Pathology, Tata Memorial Hospital, Parel, Mumbai, India

## Abstract

**Background:**

We report a case of vascular malformation arising from internal jugular vein presenting as mass at root of neck with no clinical stigmata which to the best of our knowledge is the first reported case of an intrinsic vascular malformation arising from the internal jugular vein. Magnetic resonance imaging features of this new entity have been described.

**Case presentation:**

A 27 year male presented with a gradually enlarging, asymptomatic swelling on left supraclavicular region with normal overlying skin. A soft mass, about 7 × 7 cm with restricted mobility was found with normal cranial nerve function. Fine needle aspiration cytology showed a hemorrhagic aspirate. Doppler showed a mass displacing left carotid artery posteriorly while left internal jugular vein was not visualized. Magnetic resonance imaging showed a well defined mass isointense to hypointense on T1 weighted and hyperintense on T2 weighted and STIR images with fluid-fluid levels. On exploration, a vascular mass arising from left internal jugular vein was found with good tissue planes, which was excised after ligating the patent internal jugular vein above and below the lesion. Histopathologic examination confirmed the diagnosis of vascular malformation.

**Conclusion:**

The diagnosis of intrinsic vascular malformation arising from internal jugular vein should be kept in differential while dealing with masses at root of neck and magnetic resonance imaging features may help in the pre-operative diagnosis of this entity.

## Background

Vascular lesions have been classified as hemangiomas or vascular malformations depending on the presence of cellular proliferation [1]. Venous vascular malformations may present as isolated neck masses in adults without the clinical findings typically associated with vascular malformations like skin discolouration, dilated subcutaneous vessels and compressibility [2]. Among the vessels of jugular system in the neck, intrinsic vascular malformations have been described arising from the external jugular vein [3,4]. We report a case of intrinsic vascular malformation arising from internal jugular vein presenting as mass at the root of neck with clinical and radiological features suggesting a mass in relation with major vessels of neck. Final diagnosis was established only after surgical excision and histopathology. This is the first reported case of an intrinsic vascular malformation arising from the internal jugular vein to the best of our knowledge.

## Case presentation

A 27-year male presented with a gradually enlarging mass in left supraclavicular area for the last three years. There were no associated symptoms. On physical examination, there was a mass about 7 × 7 cm, soft and non tender on the left side of neck extending from mid neck down to the clavicle. Overlying skin was normal in colour and texture. There were no pulsations or bruit over the mass. Mass showed restricted side to side mobility but was not mobile in supero-inferior direction. There was no other palpable mass or lymph node detected in the rest of the neck. Examination of all the cranial nerves was normal. Doppler of the neck vessels showed that the left common carotid artery was displaced posteriorly by a supraclavicular mass. The mass closely abutted the artery and showed indistinct fat planes with the vessel wall at places but there was no evidence of luminal narrowing noted in the course of carotid artery. The common carotid artery, external and internal arteries showed normal colour, flow and velocity. The left internal jugular vein was not visualized.

**Figure 1:**
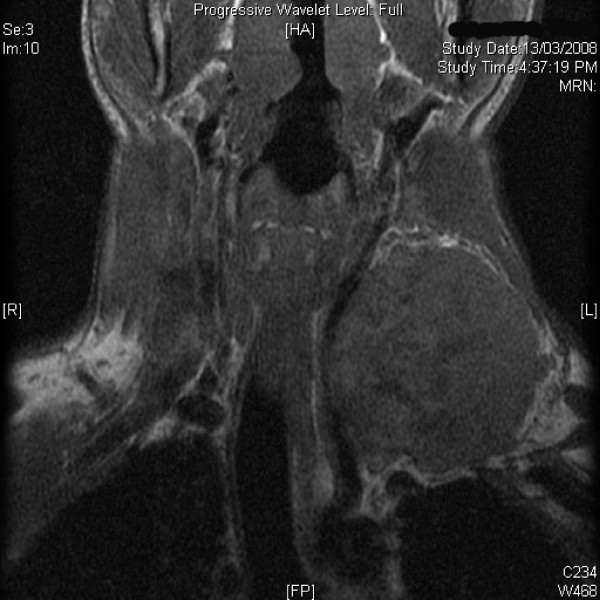
**T1 W magnetic resonance image of the neck (coronal section) showing a mass in infrahyoid neck which is isointense to hypointense to muscle**.

**Figure 2:**
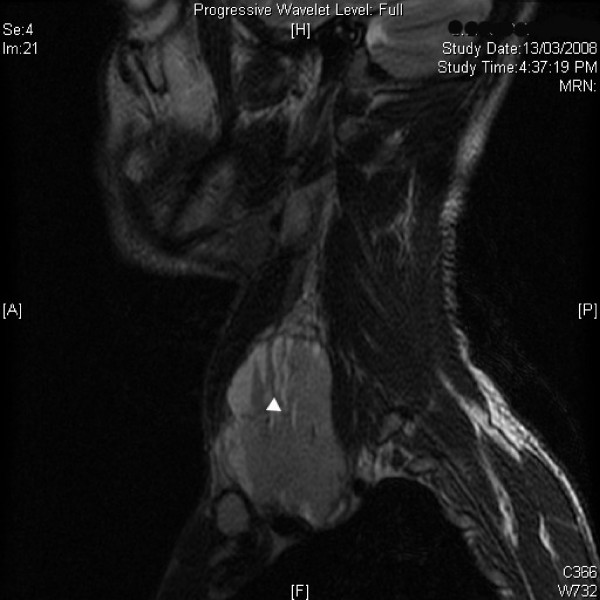
**T2 W magnetic resonance image of the neck (sagital section) showing hyperintense mass with fluid-fluid levels (arrow head, pointing upwards)**.

**Figure 3:**
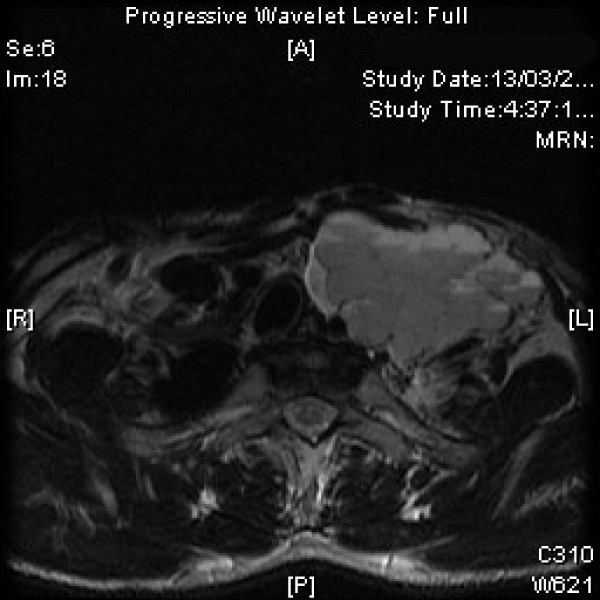
**T2 W magnetic resonance image of the neck (axial section) showing hyperintense mass with fluid-fluid levels**.

**Figure 4:**
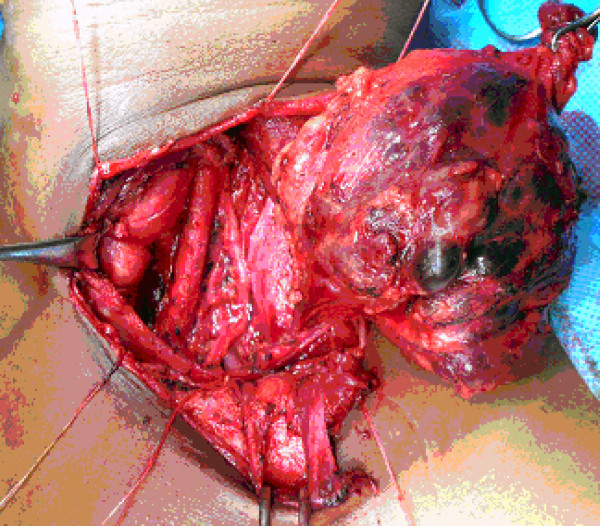
**intra-operative photograph showing left internal carotid artery, vagus nerve and vascular mass involving the left internal jugular vein**.

**Figure 5:**
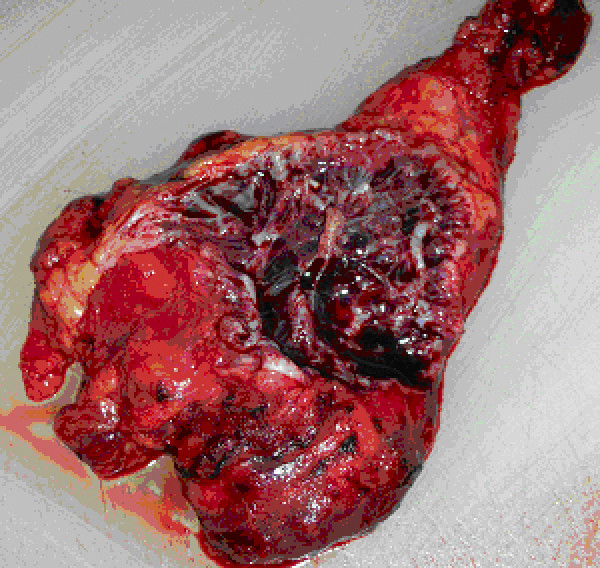
**gross cut section of the specimen showing multiple vascular channels**.

**Figure 6:**
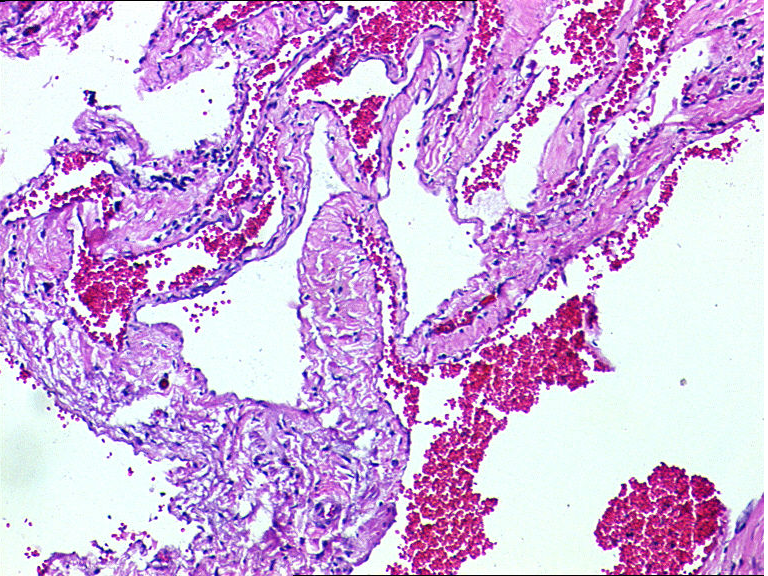
**Histological slide of the mass showing dilated vascular channels of vascular caliber with thick muscular walls and focal calcification**.

**Figure 7:**
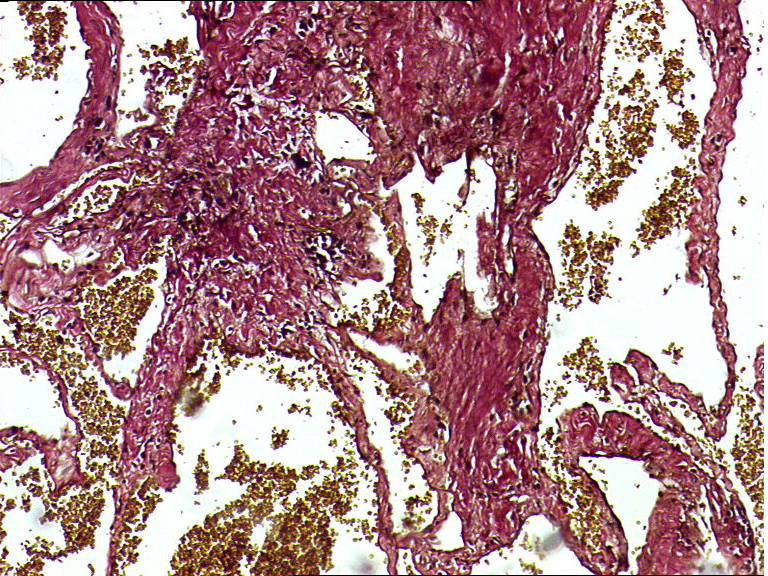
**Elastin vangeison (EVG) stained section showing lack of elastic fibers reinforcing venous origin**.

Magnetic resonance imaging (MRI) demonstrated a heterogenous well defined mass, 7 × 4 × 6 cm in size in infra hyoid neck on the left side. The lesion appeared isointense to hypointense to muscle on T1 weighted images [Figure 1] and hyperintense on T2 weighted [Figure 2, 3] and STIR images with fluid-fluid level. The left internal jugular vein superior to the mass was well defined and at the level of the mass was compressed. There was hyperintense signal in the left internal jugular vein secondary to flow related phenomenon. Fine needle aspiration cytology (FNAC) of the mass showed a hemorrhagic aspirate and bleeding from the puncture site stopped with local pressure for few minutes. The patient was planned for exploration and excision of the mass as an elective procedure. Intra-operatively, a mass arising from the lower part of left internal jugular vein was identified which was pushing the common carotid artery and vagus nerve (Figure 4). These were dissected off the mass as good tissue planes were available around the mass. The proximal and distal ends of the internal jugular vein were ligated and the mass was completely excised. Gross cut section of the mass showed multiple dilated vascular channels [Figure 5]. Histopathology revealed many dilated, ectatic vascular channels with muscular walls [Figure 6, Figure 7]. The intervening fibroadipose tissue showed scattered chronic inflammatory cells and focal calcification consistent with the diagnosis of vascular malformation.

## Discussion

Intrinsic vascular malformations arising from the wall of internal jugular vein have not been described in English-language literature. Few cases of intrinsic vascular malformations arising from external jugular vein have been reported [3,4]. We describe a case of vascular malformation of internal jugular vein which presented in an adult as a slowly enlarging mass at the root of neck with no other signs and symptoms, for which the diagnosis was established after surgical exploration and histopathology.

Venous vascular malformations are believed to be present at birth but may not become apparent until later in life. They may expand as a result of trauma, sepsis, hormonal changes or changes in venous pressure [5,6]. MR features of head and neck vascular malformations typically show intermediate signal intensity (slightly higher than that of muscles) on T1 weighted images, hyperintense in T2 weighted images and variable enhancement after intravenous gadolinium administration [7]. Intrinsic vascular malformations have been reported involving the external jugular vein and having similar features. Authors recommend that this entity involving internal jugular vein should be kept in the differential of such lesions.

Similar MRI characteristics like an isointense T1 signal relative to skeletal muscle with hyperintense and slightly heterogenous T2 signal [8] have been reported in head and neck schwannomas. In the present case, the mass was asymptomatic, of long duration with radiological features similar to a cystic schwannoma. Head and neck schwannomas are uncommon nerve sheath neoplasms which may present as diagnostic and management challenges. Majority of extra-cranial schwannomas present as asymptomatic stable neck masses of long standing duration that caused little concern other than the possibility of malignancy and cosmesis [9]. Parapharyngeal space is the most common site for these tumours [10,11] but they can present lower down in the neck [9].

The root of the neck is an uncommon location for a soft compressible swelling. Lymph node enlargement can occur in this region but will be firmer in consistency and very well defined. Various swellings, such as intramuscular hemangioma, lymphangioma, lipoma, rhabdomyoma are to be considered in the differential diagnosis[12]. Also, other primary tumours like low-grade malignant hemangioendothelioma, hemangiopericytoma and frankly malignant hemangiosarcoma and leiomyosarcoma may be considered in the differential of these vascular lesions. These tumours are all very rare but may enter the differential list if the lesion shows evidence of enlargement or local infiltration clinically or on imaging studies [4].

In the present case, FNAC was performed pre-operatively but surgical excision rather than a guided biopsy is preferable because of the possibility of extensive bleeding of the punctured vascular lesion [3]. Total excision allows a better histopathologic definition and is curative as has been seen in the present case.

## Conclusion

This is first reported case of an intrinsic vascular malformation of internal jugular vein for which the diagnosis was possible only after surgical excision. This case report underlines the need for head and neck surgeons to be aware of such an entity and should be considered in differential diagnosis for neck masses with similar features.

## Competing interests

The authors declare that they have no competing interests.

## Authors' contributions

DP: was part of surgical team, designed the case report, carried out the literature research and manuscript preparation. CP: was part of the surgical team, guided in drafting the article and revising it critically for important intellectual content. PSP: carried out the literature research, manuscript editing and manuscript review. ND: was part of the surgical team, carried out the manuscript editing and manuscript review. JSL: helped in the radiological aspects of the present case and carried out the manuscript editing. RB: helped in the pathological aspects of the present case and carried out the manuscript editing. All the authors read and approved the final manuscript.

## Pre-publication history

The pre-publication history for this paper can be accessed here:


